# Appendiceal Foreign Bodies in Adults: A Systematic Review of Case Reports

**DOI:** 10.7759/cureus.40133

**Published:** 2023-06-08

**Authors:** Hashim E Elmansi Abdalla, Hussameldin M Nour, Muhammad Qasim, Abdul Malik Magsi, Muhammad S Sajid

**Affiliations:** 1 Department of General Surgery, New Cross Hospital, Wolverhampton, GBR; 2 Department of Digestive Disease and General Surgery, Royal Sussex County Hospital, Brighton, GBR

**Keywords:** gastro-intestinal, adult, foreign body, appendicitis, appendix

## Abstract

Adults can accidentally swallow foreign bodies (FBs) with food. In rare occasions, these can lodge in the appendix lumen causing inflammation. This is known as foreign body appendicitis. We conducted this study to review different types and management of appendiceal FBs.

A comprehensive search on PubMed, MEDLINE, Embase, Cochrane Library and Google Scholar was performed to detect appropriate case reports for this review. Case reports eligible for this review included patients above 18 years of age with all types of FB ingestion causing appendicitis. A total of 64 case reports were deemed to be eligible for inclusion in this systematic review. The patient mean age was 44.3 ± 16.7 years (range, 18-77). Twenty-four foreign bodies were identified in the adult appendix. They were mainly lead shot pellet, fishbone, dental crown or filling, toothpick, and others. Forty-two percent of the included patients presented with classic appendicitis pain, while 17% were asymptomatic. Moreover, the appendix was perforated in 11 patients. Regarding modalities used for diagnosis, computed tomography (CT) scans confirmed the presence of FBs in 59% of cases while X-ray only managed to detect 30%. Almost all of the cases (91%) were treated surgically with appendicectomy and only six were managed conservatively. Overall, lead shot pellets were the most common foreign body found. Fishbone and toothpick accounted for most of the perforated appendix cases. This study concludes that prophylactic appendicectomy is recommended for the management of foreign bodies detected in the appendix, even if the patient is asymptomatic.

## Introduction and background

Acute appendicitis is considered to be one of the most common causes of surgical emergencies and surgical admissions, with lifetime risk of 8.6% in males and 6.7% in females [1]. Acute appendicitis is caused by luminal obstruction leading to inflammation and infection; this can be due to conglomeration of faeces (appendicolith), calculi, lymphoid hyperplasia, benign or malignant tumours and lymphoid follicular hyperplasia secondary to infection elsewhere [2].

Alvarado score, Appendicitis Inflammatory Response Score (AIRS) and the Adult Appendicitis Score are all known scoring systems used to help stratify patients into low risk and high risk for acute appendicitis aiming to guide management [3]. Furthermore, radiological investigations such as ultrasound (US) and computed tomography (CT) are widely used to diagnose appendicitis. CT is considered the best method in detecting acute appendicitis in suspected cases; however, it carries the burden of radiation. Therefore, US can also be a useful tool to diagnose appendicitis; however, US sensitivity is only 83.7% and it depends greatly on the operator [4]. Also, despite the emerging evidence of using antibiotics for the treatment of acute appendicitis, surgical removal of the acutely inflamed appendix is still considered the gold standard management of acute appendicitis [1].

Adults can inadvertently ingest foreign bodies (FBs) while eating, the most common being fish bones and chicken bones. They can cause symptoms depending on their composition and where they lodge in the intestinal tract [5]. Rarely, they can lodge in the lumen of the appendix causing inflammation, obstruction and possibly perforation of the appendix [6]. The aim of this study was to conduct a literature review highlighting various types of foreign bodies causing acute appendicitis, presentation, mode of diagnosis and management in the adult population.

## Review

Methods

Information Extraction Method

To identify appropriate case reports for this review, a comprehensive search strategy was conducted across PubMed, MEDLINE, Embase, and the Cochrane Library. Using the Medical Subject Headings (MeSH) search terms found in the MEDLINE library, the relevant case reports were clustered based on their association with FB appendicitis in adults. A combination of Boolean operators (AND, OR, NOT) was used to narrow down and widen the search results. In addition to the selected reports, references were reviewed as an additional source of literature.

Inclusion/Exclusion Criteria

Case reports were considered eligible if patients were 18 years old or above with all types of FB ingestion causing appendicitis. All genders and ethnicities were included. The literature review also included case reports published in other languages if the translated into English abstract had the required data, which consisted of first author's last name, country and year of the publication, age, gender, type of FB ingested, mode of investigation and treatment; otherwise, they were excluded.

Data Collection and Analysis

The title, abstract and full text of case reports were examined by three different authors on a pre-agreed upon extraction sheet. It was compared and found to be in rational agreement. Upon conclusion of data extraction, a mutual consensus was reached between the different examiners after a detailed discussion.

Quality of the case reports was assessed using the Joanna Briggs Institute (JBI) critical checklist for appraisal [7]. Statistical analysis of the data variables in this study was descriptive and performed with Microsoft Excel 2019, version 16 (Microsoft, Redmond, WA). For quantitative variables, summary measures including mean, standard deviation, median, and maximum and minimum values were calculated. Data corresponding to the qualitative variables were assessed as absolute and relative (percentage) frequencies.

Results

**Figure 1:**
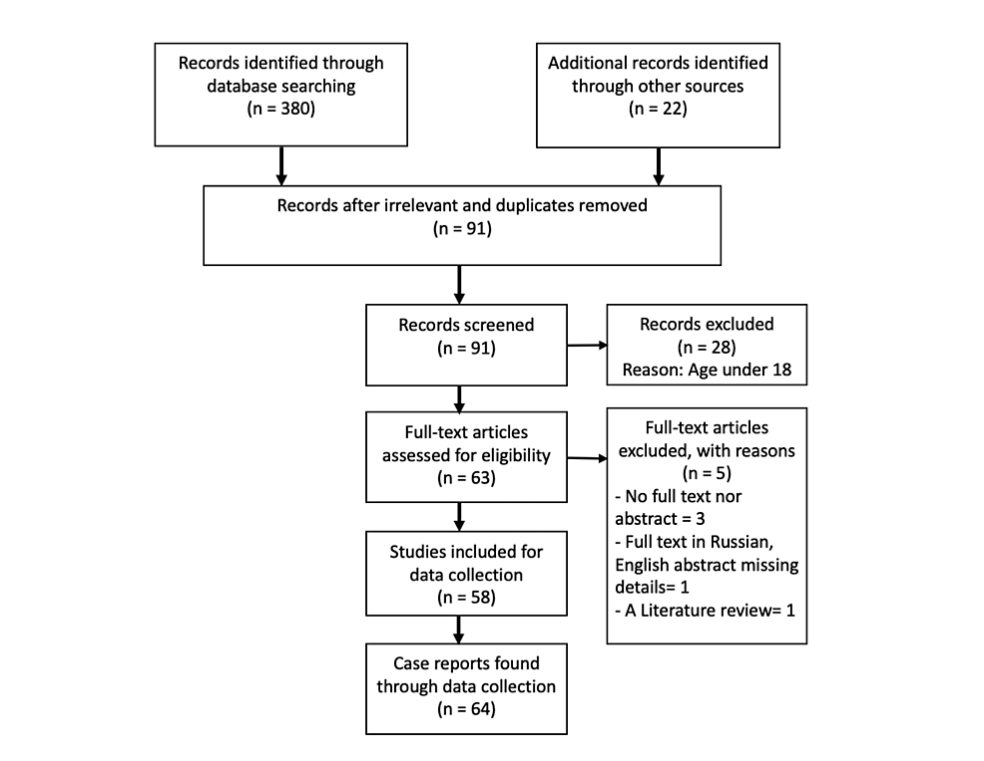
Preferred Reporting Items for Systematic Reviews and Meta-Analyses (PRISMA) flow chart showing literature search outcomes for the systemic review Additional resources: references of the reported cases or reviews

After removal of ineligible publications, the literature search produced one case series and literature review study reporting 7 cases [8] and 57 case reports [5,6,9-64]. One Russian case series study that only mentioned the number of cases in abstract but not full details was excluded, and 27 reporting cases in under 18 were excluded as well [65]. Details of our systematic search are shown in Figure 1.

On the retrospective review of 64 case reports [5,8-64] (from 1971 to 2022) describing appendicitis induced by foreign body and fulfilling the inclusion criteria, we recorded the patient age, gender, type of foreign body, diagnostic imaging identifying the FB in the appendix, and management. Cases were reported from 25 countries, with 18 cases reported from USA [8-19], seven from UK [5,20-25], five from Japan [26-30] and Korea [31-35] each, three from the Netherlands [36-38] and from Turkey [39-41]. Australia [42,43], Ecuador [44,45], Italy [46,47] and Taiwan [48,49] each had two cases while the rest of the following reported only one case: Austria, Cameroon, China, Croatia, France, Germany, Iran, Ireland, Kuwait, Morocco, New Zealand, Peru, Saudi Arabia, Singapore and Spain [50-64]. Table 1 illustrates characteristics of case reports (author, year, country, age and gender).

**Table 1:** Case report demographics

Reference	Author and year	Country	Age	Gender
[8]	Balch and Silver, 1971	USA	20	Male
[8]	Balch and Silver, 1971	USA	25	Female
[8]	Balch and Silver, 1971	USA	34	Male
[8]	Balch and Silver, 1971	USA	40	Female
[8]	Balch and Silver, 1971	USA	40	Female
[8]	Balch and Silver, 1971	USA	66	Male
[8]	Balch and Silver, 1971	USA	40	Female
[9]	Bababekov et al., 2015	USA	63	Male
[10]	Brennan et al., 2022	USA	51	Male
[11]	Cheng et al., 2021	USA	23	Female
[12]	Christakis et al., 2011	USA	57	Male
[13]	Cox and Pesola, 2005	USA	73	Female
[14]	Green et al., 1994	USA	27	Male
[15]	Mallory et al., 2004	USA	59	Male
[16]	Meyer et al., 1982	USA	25	Male
[17]	Palmer and Shortsleeve, 1998	USA	42	Male
[18]	Pagacz et al., 2020	USA	49	Female
[19]	Song et al., 2009	USA	53	Male
[5]	Mellor and Mellor, 1994	UK	48	Male
[20]	Basu et al., 2009	UK	75	Male
[21]	Glen et al., 2007	UK	41	Male
[22]	Munipalle et al., 2013	UK	45	Male
[23]	Packard et al., 2019	UK	60	Male
[24]	Sar et al., 2009	UK	70	Male
[25]	Sian and Lloyd, 2003	UK	45	Male
[26]	Adachi et al., 2014	Japan	57	Male
[27]	Hoshino et al., 2010	Japan	66	Female
[28]	Tanaka et al., 2007	Japan	77	Female
[29]	Tsukamoto et al., 2018	Japan	40	Male
[30]	Uchihara et al., 2022	Japan	50	Male
[31]	Asad et al., 2007	Korea	39	Male
[32]	Baek et al., 2012	Korea	54	Female
[33]	Ha et al., 2009	Korea	34	Male
[34]	Kim et al., 2015	Korea	22	Male
[35]	Rim and Song, 2019	Korea	31	Male
[36]	Bouwman and van der Made, 2006	Netherlands	24	Female
[37]	Hoogstad Evert et al., 2021	Netherlands	35	Female
[38]	van den Berg et al., 1999	Netherlands	49	Male
[39]	Abdullazade et al., 2017	Turkey	27	Male
[40]	Hazer et al., 2013	Turkey	20	Male
[41]	Ozkan et al., 2015	Turkey	45	Male
[42]	Cui et al., 2018	Australia	25	Male
[43]	Smith et al., 2017	Australia	67	Male
[44]	Aguilar Ayala et al., 2019	Ecuador	27	Male
[45]	Cevallos et al., 2019	Ecuador	27	Male
[46]	Antonacci et al., 2013	Italy	45	Male
[47]	Grassi et al., 2016	Italy	65	Male
[48]	Chou et al., 2016	Taiwan	54	Male
[49]	Lin and Wu, 2014	Taiwan	54	Male
[50]	Renner et al., 2000	Austria	26	Male
[51]	Sama et al., 2016	Cameroon	26	Female
[52]	Price et al., 1988	China	22	Male
[53]	Perko et al., 2008	Croatia	69	Male
[54]	Benizri et al., 2012	France	29	Female
[55]	Comman et al., 2008	Germany	54	Female
[56]	Dehghan et al., 2011	Iran	50	Female
[57]	Lloyd et al., 2022	Ireland	64	Female
[58]	Qassim et al., 2021	Kuwait	40	Female
[59]	Harhar et al., 2021	Morocco	18	Female
[60]	Field et al., 2022	New Zealand	52	Male
[61]	Casas Roca et al., 2022	Peru	26	Male
[62]	Khazindar et al., 2021	Saudi Arabia	19	Male
[63]	Beh et al., 2016	Singapore	72	Male
[64]	Rodríguez Lucas et al., 2022	Spain	50	Female

The mean age of patients was 44.3 ± 16.7 years, ranging from 18 (58) to 77 (27), with gender distribution favouring male patients (44:20). Twenty-four different foreign bodies were identified including lead shot pellet (19%), dental crown/filling (11%), dental metallic FB (9.3%), fishbone (6.3%), metallic FB (6.3%), toothpick (6.3%), bone fragment (5%), needle (5%), finger nail (3%), metal nail (3%), mercury (3%) and pin (3%), and the rest were identified once each (airgun pellet, ballerine intra-uterine device, or IUD, coin battery, endodontic file, endoluminal clip, hair follicle, jackstone, razor blade, rubber FB, seed, tongue piercing and trichobezoar).

Out of 64 cases, the majority (42%) presented to the hospital with the classic appendicitis pain in the right lower abdomen; 20 patients had vague peri-umbilical or lower abdominal pain with four of them having associated fever, one feeling fatigued and another with loss of appetite. Eleven were asymptomatic presenting only due to FB ingestion. Imaging was done to detect the FB location; once found in the appendix, the patient was taken to the operating theatre for FB retrieval to prevent complications and turned out to have incidental diagnosis of foreign body-induced appendicitis or it was found upon examination of the specimen later on. Perforated appendix causing peritonitis was seen in four patients. Perforation of the appendix with or without peritonitis was seen in 10 (16%) cases with fishbone and toothpick being the main culprits (50%). Clinical presentations and perforation rates for different foreign body types are illustrated in Table 2.

**Table 2:** Clinical presentations and perforation rates for different FB types FB, foreign body; IUD, intra-uterine device *Sharp or pointed-edge object

FB type	(n)		Right iliac fossa pain	Vague lower abdominal pain			Asymptomatic	Peritonitis	Perforation
				Pain only	+ Fever	+ Vomiting			
Lead shot pellet		(12)	5 (42%)	3 (25%)	0	0	3 (25%)	1 (8%)	1 (8%)
Dental crown/filling		(7)	3 (43%)	2 (29%)	0	0	1 (14%)	1 (14%)	1 (14%)
Dental metallic FB*		(6)	2 (33.3%)	1 (16.7%)	1 (16.7%)	0	2 (33.3%)	0	1 (16.7%)
Fishbone*		(4)	4 (75%)	0	0	0	0	0	3 (75%)
Metallic FB		(4)	4 (100%)	0	0	0	0	0	0
Needle*		(4)	0	1 (25%)	0	0	2 (50%)	1 (25%)	1 (25%)
Toothpick*		(4)	1 (25%)	1 (25%)	1 (25%)	1 (25%)	0	0	2 (50%)
Bone fragment*		(3)	0	3 (100%)	0	0	0	0	0
Pin*		(2)	0	1 (50%)	0	1 (50%)	0	0	0
Metal nail*		(2)	0	0	0	0	2 (100%)	0	0
Finger nail*		(2)	2 (100%)	0	0	0	0	0	0
Mercury		(2)	1 (50%)	0	0	0	0	1 (50%)	1 (50%)
Airgun pellet		(1)	0	0	1 (100%)	0	0	0	0
Ballerine IUD		(1)	0	1 (100%)	0	0	0	0	0
Coin battery		(1)	0	0	1 (100%)	0	0	0	0
Endodontic file*		(1)	0	1 (100%)	0	0	0	0	0
Endoluminal clip		(1)	1 (100%)	0	0	0	0	0	0
Hair follicle		(1)	1 (100%)	0	0	0	0	0	0
Jackstone		(1)	1 (100%)	0	0	0	0	0	0
Razor blade*		(1)	1 (100%)	0	0	0	0	0	0
Rubber FB – condom		(1)	1 (100%)	0	0	0	0	0	0
Seed		(1)	0	0	0	0	1 (100%)	0	0
Tongue piercing		(1)	1 (100%)	0	0	0	0	0	0
Trichobezoar		(1)	1 (100%)	0	0	0	0	0	0
Total		(64)	29 (45%)	14 (22%)	4 (6%)	2 (3%)	11 (17%)	4 (6%)	10 (16%)
				20 (33%)					

The main diagnostic imaging modality to detect foreign bodies was CT of the abdomen and pelvis as reported in 58% of cases; almost half of those who went to have a CT scan had a suspicious X-ray radiograph in the first instance and one also had a US scan that was normal. Diagnosis made using only X-ray was reported in 30%, and incidental identification of foreign body intra-operatively in patients presenting with appendicitis or at specimen examination accounted for seven (11%) cases. Definitive diagnosis of the FB in the appendix is shown in Table 3.

**Table 3:** Definitive diagnosis of the FB in the appendix FB, foreign body; IO, intra-operatively; POS, post-operative specimen; IUD, intra-uterine device *Sharp or pointed-edge object

FB type	(n)	X-ray	CT scan	Incidental (IO or POS)
Lead shot pellet	(12)	7 (58.3%)	3 (25%)	2 (17%)
Dental crown/filling	(7)	1 (14%)	6 (86%)	0
Dental metallic FB*	(6)	2 (33.3%)	4 (66.7%)	0
Fishbone*	(4)	0	4 (100%)	0
Metallic FB	(4)	1 (25%)	3 (75%)	0
Needle*	(4)	1 (25%)	3 (75%)	0
Toothpick*	(4)	0	2 (50%)	2 (50%)
Bone fragment*	(3)	0	3 (100%)	0
Pin*	(2)	1 (50%)	1 (50%)	0
Metal nail*	(2)	0	2 (100%)	0
Finger nail*	(2)	1 (50%)	1 (50%)	0
Mercury	(2)	1 (50%)	1 (50%)	0
Airgun pellet	(1)	1 (100%)	0	0
Ballerine IUD	(1)	0	0	1 (100%)
Coin battery	(1)	0	1 (100%)	0
Endodontic file*	(1)	1 (100%)	0	0
Endoluminal clip	(1)	0	1 (100%)	0
Hair follicle	(1)	0	1 (100%)	0
Jackstone	(1)	1 (100%)	0	0
Razor blade*	(1)	0	1 (100%)	0
Rubber FB – condom	(1)	0	0	1 (100%)
Seed	(1)	1 (100%)	0	0
Tongue piercing	(1)	0	1 (100%)	0
Trichobezoar	(1)	0	0	1 (100%)
Total	(64)	19 (30%)	38 (59%)	7 (11%)

In regard to managing FB-induced appendicitis, most of the cases were operated on (91%) with 27 patients undergoing laparoscopic appendicectomy, 15 open appendicectomies, 15 laparotomies and one recent robotic-assisted laparoscopic appendicectomy, leaving only four patients managed conservatively and two through colonoscopy in an attempt to retrieve the foreign body endoscopically. Management approaches for the various types of foreign bodies found in the appendix are presented in Table 4.

**Table 4:** Management of various foreign bodies found in the adult appendix FB, foreign body; IUD, intra-uterine device *Sharp or pointed-edge object

FB type	(n)		Surgery				Colonoscopy	Conservative
			Laparoscopic appendicectomy	Open appendicectomy	Laparotomy	Robotic		
Lead shot pellet		(12)	3 (25%)	2 (17%)	4 (33.3%)	0	0	3 (33.3%)
Dental crown/filling		(7)	2 (28.6%)	2 (28.6%)	2 (28.6%)	1 (14%)	0	0
Dental metallic FB*		(6)	3 (50%)	1 (16.7%)	0	0	1 (16.7%)	1 (16.7%)
Fishbone*		(4)	3 (75%)	1 (25%)	0	0	0	0
Metallic FB		(4)	0	2 (50%)	2 (50%)	0	0	0
Needle*		(4)	3 (75%)	0	1 (25%)	0	0	0
Toothpick*		(4)	3 (75%)	1 (25%)	0	0	0	0
Bone fragment*		(3)	1 (33.3%)	0	2 (66.7%)	0	0	0
Pin*		(2)	1 (50%)	0	1 (50%)	0	0	0
Metal nail*		(2)	0	1 (50%)	0	0	1 (50%)	0
Finger nail*		(2)	2 (100%)	0	0	0	0	0
Mercury		(2)	1 (50%)	1 (50%)	0	0	0	0
Airgun pellet		(1)	0	0	1 (100%)	0	0	0
Ballerine IUD		(1)	1 (100%)	0	0	0	0	0
Coin battery		(1)	1 (100%)	0	0	0	0	0
Endodontic file*		(1)	1 (100%)	0	0	0	0	0
Endoluminal clip		(1)	0	0	1 (100%)	0	0	0
Hair follicle		(1)	0	1 (100%)	0	0	0	0
Jackstone		(1)	0	0	1 (100%)	0	0	0
Razor blade*		(1)	1 (100%)	0	0	0	0	0
Rubber FB – condom		(1)	0	1 (100%)	0	0	0	0
Seed		(1)	0	1 (100%)	0	0	0	0
Tongue piercing		(1)	1 (100%)	0	0	0	0	0
Trichobezoar		(1)	0	1 (100%)	0	0	0	0
Total		(64)	27 (42%)	15 (23%)	15 (23%)	1 (2%)	2 (3%)	4 (6%)
			58 (91%)					

Discussion

To our knowledge, this is the first and largest focused systematic review of case reports describing appendiceal foreign bodies in adults. In this study, most of the FB-induced appendicitis adult case reports were published in the last two decades (75%) with the most common reported FB being lead shot pellet (12 of 64, 19%), although in the last decade, dental FBs were found to be more common (8 out of 29, 27.5%). In comparison, Balch and Silver who reviewed cases from all age groups up to 1971 concluded that pins were the most common FB found in the appendix, followed by lead shot pellets and then bones [8]. Meanwhile, hair was the most common FB found in paediatric appendix cases according to Fuller et al. [66]. They also observed that hair was also the commonest cause of FB-related appendix perforation in children while that was not the case in our study as fishbone (3 of 10 perforations, 30%) was the commonest in adults.

Historically, the incidence of FB being found in the lumen of the appendix following appendicectomy was found to be 3% and 0.0005% in 1963 by Collins and 1971 by Balch and Silver, respectively [8]. Adults can accidentally swallow foreign bodies with food; in rare occasions, they can lodge in the appendix lumen, causing inflammation and in some cases perforation. This is known as foreign body appendicitis [5,6]. The first reported foreign body appendicitis was in an 11-year-old child back in 1735 when a pin was found perforating the appendix of the child undergoing appendicectomy by the French surgeon Claudius Amyand in London [8]. Since then, there have been many cases reported with the majority being before the turn of the 20th century and it is thought to be related to accidentally swallowing sewing needles or pins, and also eating animals who were hunted and shot with lead pellets [67]. Ingested lead shot pellets were common among Eskimo groups and as reported by Reddy in 1985, 62 Eskimos were followed up for lead shots found radiographically in their appendix and none of them actually developed symptoms of appendicitis, but shot pellet accumulation can result in perforation or lead poisoning [68].

Balch and Silver were able to describe and collect cases backdated as far as possible, and by 1971, a total of 225 cases were reported in all age groups [8]. In 1998, Klingler et al. published a 100-year literature review for foreign bodies found in the appendix and the study was able to identify another 31 cases, increasing the total to 256 by the turn of the 21st century [67]. Since then, 53 adult case reports have been published as demonstrated in our study, excluding the cases covered by Balch and Silver [8] and Klingler [67] study, one Russian study [65] that was excluded from our review (due to lack of details) reporting eight cases, as well as the 27 paediatric cases that were also excluded, in addition to the multi-institutional case series published by Fuller et al. in 2022 describing 56 cases of FB appendicitis in the paediatric population [66]. Thus, to the best of our knowledge and efforts, this brings the total number of foreign body appendicitis cases reported in the history to at least 400 cases.

Our study reported that seven (11%) cases of foreign bodies found in the appendix were in fact found incidentally in patients undergoing surgery for classic acute appendicitis presentation. But, in general, the accurate detection of foreign bodies in the adult appendix was made possible by the use of CT in majority of patients (38 of 64, 59%). Of these 38 patients, half had a plain radiograph (X-ray) beforehand, but they were unable to either accurately describe the FB or its location. X-ray imaging was used to detect FBs, but mainly two decades ago, it became less reliable as it cannot clearly pinpoint where the FB is and can only show objects depending on their radioparency. These results support proceeding to CT as definitive imaging when patients present with abdominal symptoms but generally for FB ingestion. The literature recommends doing X-ray first as it is cost-effective and has less radiation effects on the patient but to directly perform a CT scan if sharp objects are suspected, such as fishbone and chicken bone or hair bezoar, to accurately locate them and detect their complications, which are perforation and obstruction [69].

With regard to managing FB-induced appendicitis, we found that surgery was the mainstay approach (91%). Seven out of the 58 patients who were managed surgically were initially given a trial of non-surgical treatment in the form of bowel prep [15,35], enteroscopy [23], colonoscopy [15,28], conservatively with intravenous antibiotics [24], trial with laxatives and liquid diet [50], and serial X-ray follow-up [24], but unfortunately they all failed. There were also successful non-surgical approaches seen in six cases (9%); four patients were treated conservatively, some with laxatives and others with just a serial follow-up, and the last two were successfully managed using colonoscopy.

Although ingested foreign bodies most likely pass uneventfully through the gastrointestinal tract, this is not always true for sharp or pointed objects as they have a higher rate of appendix perforation (70%). Sometimes non-sharp and more rounded objects can also cause perforation as seen in three cases related to lead shot pellet, mercury and a tooth. Balch and Silver recommend prophylactic appendicectomy for pointed objects lodged in the appendix while Klinger et al. recommend attempting gastroscopy or colonoscopy before resorting to laparoscopic-guided removal [8,67]. Unfortunately, the latter recommendation would not be applicable in most cases; hence, the results support offering prophylactic appendicectomy especially if sharp objects were identified, even if patients are asymptomatic as there would still be evidence of inflammation. The aim is to prevent severe and fatal complications. In some cases with less risky foreign bodies, endoscopy or conservative management for spontaneous passage may be the option.

Limitations

Being a systematic review of published case reports, we were unable to retrieve detailed records of foreign body appendicitis adult cases before 1971, as these case reports were not published electronically/online. Another limitation is that, to our knowledge, there has not been any retrospective or prospective study conducted.

## Conclusions

Lead shot pellets are the most common FBs identified in the adult appendix leading to foreign body-induced appendicitis, with a recent uptrend of dental FBs. In the cases published, a high percentage required surgery and had accompanied perforation. Offering symptomatic and asymptomatic patients prophylactic appendicectomy when sharp or pointed objects are identified is the recommended treatment approach, to prevent severe and fatal complications. The literature review of available sources concluded that at least 400 cases of foreign body appendicitis in all age groups have been reported to date.

## References

[1] Snyder MJ, Guthrie M, Cagle S (2018). Acute appendicitis: efficient diagnosis and management. Am Fam Physician.

[2] Moris D, Paulson EK, Pappas TN (2021). Diagnosis and management of acute appendicitis in adults: a review. JAMA.

[3] Bom WJ, Scheijmans JC, Salminen P, Boermeester MA (2021). Diagnosis of uncomplicated and complicated appendicitis in adults. Scand J Surg.

[4] Shogilev DJ, Duus N, Odom SR, Shapiro NI (2014). Diagnosing appendicitis: evidence-based review of the diagnostic approach in 2014. West J Emerg Med.

[5] Mellor TK, Mellor SG (1995). Foreign bodies of dental origin in the appendix. J R Army Med Corps.

[6] Mohammed AA, Ghazi DY, Arif SH (2019). Ingested metallic foreign body impacted in the vermiform appendix presenting as acute appendicitis: case report. Int J Surg Case Rep.

[7] Moola S, Munn Z, Tufanaru C (2020). Chapter 7: Systematic reviews of etiology and risk. JBI Manual for Evidence Synthesis.

[8] Balch CM, Silver D (1971). Foreign bodies in the appendix. Report of eight cases and review of the literature. Arch Surg.

[9] Bababekov YJ, Stanelle EJ, Abujudeh HH, Kaafarani HM (2015). Fishbone-induced perforated appendicitis. BMJ Case Rep.

[10] Brennan ZJ, Young G, Packer K (2022). A tooth decaying in the appendix: an unusual cause of appendicitis. Cureus.

[11] Cheng He R, Nobel T, Greenstein AJ (2021). A case report of foreign body appendicitis caused by tongue piercing ingestion. Int J Surg Case Rep.

[12] Christakis A, Gandolfl A, Lavy D, Joseph S (2011). Appendiceal sequestration of ingested mercury as a cause of appendicitis. Am Surg.

[13] Cox WM, Pesola GR (2005). Buckshot ingestion. N Engl J Med.

[14] Green SM, Schmidt SP, Rothrock SG (1994). Delayed appendicitis from an ingested foreign body. Am J Emerg Med.

[15] Mallory MA, Lee AC, Imahara S, Mohan V (2004). Removal of a nail from the appendix. Gastrointest Endosc.

[16] Meyer J, Abuabara S, Barrett J, Lowe R (1982). A bullet in the appendix. J Trauma.

[17] Palmer GM, Shortsleeve MJ (1998). Transient golden appendicolith. South Med J.

[18] Pagacz M, Bao P, Moreno JC, Howard L (2020). Nail biting as a cause of appendicitis. Case Rep Surg.

[19] Song YS, Covarrubias DA, Nardi PM (2009). Foreign body appendicitis. AJR Am J Roentgenol.

[20] Basu NN, Doddi S, Turner L, Sinha PS (2009). Tooth crown foreign body appendicitis. Dig Surg.

[21] Glen P, Ihedioha U, Mackenzie I (2007). An unusual extraction; retrieval of a swallowed crown by appendicectomy. Br Dent J.

[22] Munipalle PC, Little M, Garud T (2013). Lead shot incarceration in appendix: an unusual cause of appendicular foreign body. BMJ Case Rep.

[23] Packard E, Groff A, Shahid Z, Sahu N, Jain R (2019). A 'bit' of appendicitis: a case of a foreign object in the adult appendix. Cureus.

[24] Sar S, Mahawar KK, Marsh R, Small PK (2009). Recurrent appendicitis following successful conservative management of an appendicular mass in association with a foreign body: a case report. Cases J.

[25] Sian PS, Lloyd DM (2003). Removal of retained lead shot through laparoscopic appendectomy. JSLS.

[26] Adachi M, Takahashi Y, Kume M, Kurenuma A, Motohashi M, Muramatsu Y (2014). Barium-induced appendicitis mimicking accidental ingestion of a dental metal crown in radiological findings. Clin J Gastroenterol.

[27] Hoshino I, Sugamoto Y, Fukunaga T (2010). Appendicitis caused by an endoluminal clip. Am J Gastroenterol.

[28] Tanaka K, Toyoda H, Aoki M, Noda T, Aota T (2007). An incarcerated prosthetic tooth in the vermiform appendix. Gastrointest Endosc.

[29] Tsukamoto R, Miyano S, Machida M, Kitabatake T, Fujisawa M, Kojima K (2018). Appendiceal perforation due to migration of a dental instrument. Case Rep Gastroenterol.

[30] Uchihara T, Komohara Y, Yamashita K, Arima K, Uemura S, Hanada N, Baba H (2022). Chronic appendicitis caused by a perforating fish bone: case report and brief literature review. In Vivo.

[31] Asad S, Bae K, Jeon KN, Cho JM, Shin TB, Joo YT, Kim HJ (2007). Appendicitis caused by a foreign body of dental origin: diagnosis with ultrasonography. J Ultrasound Med.

[32] Baek SK, Bae OS, Hwang I (2012). Perforated appendicitis caused by foreign body ingestion. Surg Laparosc Endosc Percutan Tech.

[33] Ha NR, Lee HL, Yoon JH (2009). A case of an ingested sewing needle in the appendix. Gastrointest Endosc.

[34] Kim JH, Lee DS, Kim KM (2015). Acute appendicitis caused by foreign body ingestion. Ann Surg Treat Res.

[35] Rim CB, Song KH (2019). Impaction of coin battery in the appendix. Korean J Gastroenterol.

[36] Bouwman LH, van der Made WJ (2006). Diagnostic image (288). A woman with lower right abdominal pain. (Article in Dutch). Ned Tijdschr Geneeskd.

[37] Hoogstad-van Evert JS, Speelman M, Gobardhan PD (2021). A woman with abdominal pain after insertion of an intra-uterine device. (Article in Dutch). Ned Tijdschr Geneeskd.

[38] van den Berg JW, Verschuuren EA, Ouwens JP, Rottier C, Koëter GH, de Boer WJ, van der Bij W (1999). Acute abdominal pain in a lung transplant recipient. Respiration.

[39] Abdullazade S, Bayar B, Can M, Altinsoy E, Kiziloglu I (2017). Acute appendicitis due to hair follicle obstruction: a rare case report. Prz Gastroenterol.

[40] Hazer B, Dandin O, Karakaş DO (2013). A rare cause of acute appendicitis: an ingested foreign body. Ulus Travma Acil Cerrahi Derg.

[41] Ozkan OV, Muderris V, Altintoprak F, Yagmurkaya O, Yalkin O, Celebi F (2015). An unusual cause of abdominal pain: three lead pellets within the appendix vermiformis. Case Rep Surg.

[42] Cui J, Cross T, Lockwood D (2018). Ingested razor blades within the appendix: a rare case report. Int J Surg Case Rep.

[43] Smith A, Mouline O, Mallet T, Phillips G, Hartslief M (2019). Dental extraction from an appendix: a case report and review of the literature. ANZ J Surg.

[44] Aguilar Ayala BE, Cazares Cadena BR, Molina GA, Constante Ruiz JE, Salazar Parada JF, Solórzano García OJ (2019). Cecum and appendix perforation due to inadvertent ingestion of two toothpicks. J Surg Case Rep.

[45] Cevallos JM, Molina GA, Aguayo WG, Cacuango LP, Espin DS, Ramos DR, Lopez SC (2019). A nail in the appendix, accidental discovery on an asymptomatic patient. J Surg Case Rep.

[46] Antonacci N, Labombarda M, Ricci C, Buscemi S, Casadei R, Minni F (2013). A bizarre foreign body in the appendix: a case report. World J Gastrointest Surg.

[47] Grassi V, Desiderio J, Cacurri A (2016). A rare case of perforation of the subhepatic appendix by a toothpick in a patient with intestinal malrotation: laparoscopic approach. G Chir.

[48] Chou AS, Hsu YH, Wu BG (2016). Appendicular foreign body presenting with appendicular mass. Tzu Chi Med J.

[49] Lin YY, Wu RC (2014). Acute appendicitis caused by an ingested bony fragment. Intern Med.

[50] Renner K, Holzer B, Hochwarter G, Weihsbeck E, Schiessel R (2000). Needle perforation of the appendix. Dig Surg.

[51] Sama CB, Aminde LN, Njim TN, Angwafo FF III (2016). Foreign body in the appendix presenting as acute appendicitis: a case report. J Med Case Rep.

[52] Price J, Dewar GA, Metreweli C (1988). Airgun pellet appendicitis. Australas Radiol.

[53] Perko Z, Bilan K, Pogorelić Z (2008). Acute appendicitis and ileal perforation with a toothpick treated by laparoscopy. Coll Antropol.

[54] Benizri EI, Cohen C, Bereder JM, Rahili A, Benchimol D (2012). Swallowing a safety pin: report of a case. World J Gastrointest Surg.

[55] Comman A, Gaetzschmann P, Hanner T, Behrend M (2008). A case of needle ingestion in a female - laparoscopic retrieval. JSLS.

[56] Dehghan A, Moaddab AH, Mozafarpour S (2011). An unusual localization of trichobezoar in the appendix. Turk J Gastroenterol.

[57] Lloyd AJ, Abd Elwahab SM, Boland MR, Elfadul A, Hill AD, Power C (2022). Acute complicated appendicitis caused by an ingested toothpick - a case report. Int J Surg Case Rep.

[58] Qassim S, Lairy A, Asfar S (2021). Foreign body ingestion followed by appendiceal perforation. Case Rep Surg.

[59] Harhar M, Jabi R, El Harroudi T, Bouziane M (2021). Fishbone-induced appendicitis: a case report. Cureus.

[60] Field X, Burton T, Christey G (2022). Fatal sepsis from appendicitis caused by an impacted tooth. J Surg Case Rep.

[61] Casas Roca J, Ramos-Yataco A, Alcalde-Loyola C, Montalvo G, Rios-Rojas J, Bacilio Cardozo A (2022). Peritonitis due to appendicitis related to mercury sequestration: an unusual Peruvian case report. Cureus.

[62] Khazindar AR, Thabit RA, Badeeb A (2021). Bullet appendicitis: an unusual cause to a rather straight-forward diagnosis. Cureus.

[63] Beh JC, Uppaluri AS, Koh BF, Cheow PC (2016). Fishbone perforated appendicitis. J Radiol Case Rep.

[64] Rodríguez Lucas JM, Fernández López AJ, González Valverde FM, Tamayo Rodríguez ME, Albarracín Marín-Blázquez A (2022). Uncommon causes of acute appendicitis: foreign bodies in the cecal appendix. Rev Esp Enferm Dig.

[65] Davidov MI, Subbotin VM, Gerner AO, Kostarev AN, Lebedev AS, Smol'kov AA (2005). Foreign bodies of appendix and caecum complicated with acute appendicitis. (Article in Russian). Khirurgiia (Mosk).

[66] Fuller MY, Leino DG, Reyes-Múgica M (2022). Ingested foreign bodies can cause appendicitis and perforation: a multi-institutional case series. Pediatr Dev Pathol.

[67] Klingler PJ, Seelig MH, DeVault KR, Wetscher GJ, Floch NR, Branton SA, Hinder RA (1998). Ingested foreign bodies within the appendix: a 100-year review of the literature. Dig Dis.

[68] Reddy ER (1985). Retained lead shot in the appendix. J Can Assoc Radiol.

[69] Mathew RP, Sarasamma S, Jose M, Toms A, Jayaram V, Patel V, Low G (2021). Clinical presentation, diagnosis and management of aerodigestive tract foreign bodies in the adult population: Part 1. SA J Radiol.

